# Target Screen of Anti-Hyperuricemia Compounds from Cortex Fraxini In Vivo Based on ABCG2 and Bioaffinity Ultrafiltration Mass Spectrometry

**DOI:** 10.3390/molecules28237896

**Published:** 2023-12-01

**Authors:** Xiuxiu Huang, Wenqing Dong, Xiao Luo, Lu Xu, Yinan Wang

**Affiliations:** Jiangsu Key Laboratory of New Drug Research and Clinical Pharmacy, Xuzhou Medical University, 209 Tongshan Road, Xuzhou 221004, China; xxyxyxz@163.com (X.H.); jhuan0926@163.com (W.D.); luoxiao_obu1mo@163.com (X.L.)

**Keywords:** hyperuricemia, uric acid, ABCG2, ultrafiltration, Cortex Fraxini, fraxin

## Abstract

The ATP-binding cassette (ABC) transporter ABCG2 is a significant urate transporter with a high capacity, and it plays a crucial role in the development of hyperuricemia and gout. Therefore, it has the potential to be targeted for therapeutic interventions. Cortex Fraxini, a traditional Chinese medicine (TCM), has been found to possess anti-hyperuricemia properties. However, the specific constituents of Cortex Fraxini responsible for this effect are still unknown, particularly the compound that is responsible for reducing uric acid levels in vivo. In this study, we propose a target screening protocol utilizing bio-affinity ultrafiltration mass spectrometry (BA-UF-MS) to expediently ascertain ABCG2 ligands from the plasma of rats administered with Cortex Fraxini. Our screening protocol successfully identified fraxin as a potential ligand that interacts with ABCG2 when it functions as the target protein. Subsequent investigations substantiated fraxin as an activated ligand of ABCG2. These findings imply that fraxin exhibits promise as a drug candidate for the treatment of hyperuricemia. Furthermore, the utilization of BA-UF-MS demonstrates its efficacy as a valuable methodology for identifying hit compounds that exhibit binding affinity towards ABCG2 within TCMs.

## 1. Introduction

Hyperuricemia, a common disease, can arise from elevated urate synthesis or impaired renal or intestinal excretion [1]. Gout, the clinical manifestation of hyperuricemia, is precipitated by urate deposition [2]. Additionally, gout and hyperuricemia have been linked to various diseases, including chronic kidney disease, hypertension, and metabolic syndrome [3]. An effective approach to managing hyperuricemia involves inhibiting excessive uric acid (UA) production and preventing its build-up in the bloodstream, thereby maintaining a desirable level of serum uric acid (SUA). The elimination of urate primarily occurs through glomerular filtration in the kidney. The ATP-binding cassette (ABC) transporter ABCG2 has been recognized as a significant urate transporter [4,5], exhibiting high expression in both intestinal and renal epithelial cells [6,7]. ABCG2 plays a critical role in the regulation of renal urate overload and extra-renal urate underexcretion, making it a potentially valuable target for pharmacological intervention in the management of hyperuricemia or gout [8,9].

Cortex Fraxini comes from the dry bark of *Fraxinus stylosa Lingelsh* and *Fraxinus rhynchophylla Hance*, and has been utilized in China for the treatment of hyperuricemia [10]. However, the specific active compounds within Cortex Fraxini and their relationship with ABCG2 remain unknown. Since the active ingredients of traditional Chinese medicines (TCMs) are those that can be absorbed into the bloodstream following oral administration, it is imperative to identify the specific components of medicated plasma of Cortex Fraxini that can regulate ABCG2. Hence, it is imperative to identify the “effective form” of components in Cortex Fraxini that can efficiently, rapidly, and accurately activate ABCG2.

In order to expedite the process of drug discovery, various protein-ligand interaction-based methods have been devised. Among these, the integration of bioaffinity ultrafiltration and LC-MS (BA-UF-MS) has been employed to swiftly screen affinity ligands from intricate mixtures [11]. This approach proves to be time-efficient, cost-effective, and eliminates the need for intricate chemical compound isolation procedures.

**Figure 1:**
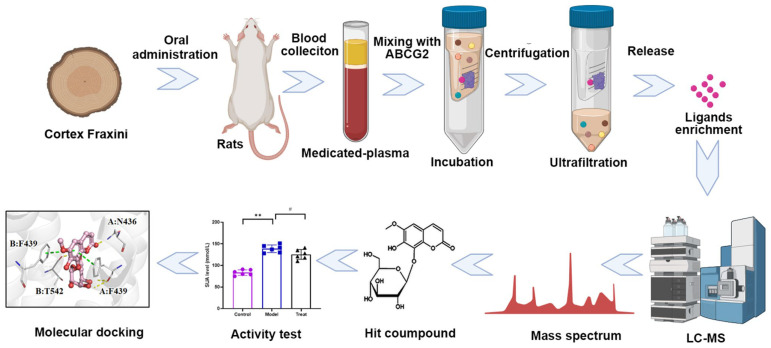
Schematic diagram of ABCG2 ligands screening with BA–UF–MS (# *p* < 0.05, ** *p* < 0.05).

This study presents an integrated approach, utilizing BA-UF-MS for the screening of potential ABCG2 ligands from medicated plasma of Cortex Fraxini. Initially, BA-UF-MS enables the swift separation and identification of the hit compound that binds to ABCG2. Subsequently, the activity of the identified hit compound is confirmed through in vitro and in vivo assays. Furthermore, the molecular docking and surface plasmon resonance (SPR) assay were employed to confirm the affinity between the hit compound and ABCG2. As a result, a potent ABCG2 activated ligand was successfully identified from the medicated plasma of Cortex Fraxini. This study represents the first attempt to utilize this integrated method for screening ABCG2 activated ligands from Cortex Fraxini. The overall research framework is depicted in Figure 1. It is anticipated that the implementation of this strategy will expedite the process of natural-product based drug discovery.

## 2. Results

### 2.1. ABCG2 Activated Assay of the Cortex Fraxini Extract

The activity of the Cortex Fraxini extract on ABCG2 was assessed, revealing a significant effect with an EC_50_ value of 35.26 mg/mL as determined by ^14^C uptake. The ABCG2 activity of the Cortex Fraxini extract had been observed, but the specific bioactive constituents responsible for affecting ABCG2 in medicated plasma of Cortex Fraxini have not been previously identified. Therefore, BA-UF-MS was employed to identify the active constituents that target ABCG2.

### 2.2. Optimization Conditions for Ligand Screen

**Figure 2:**
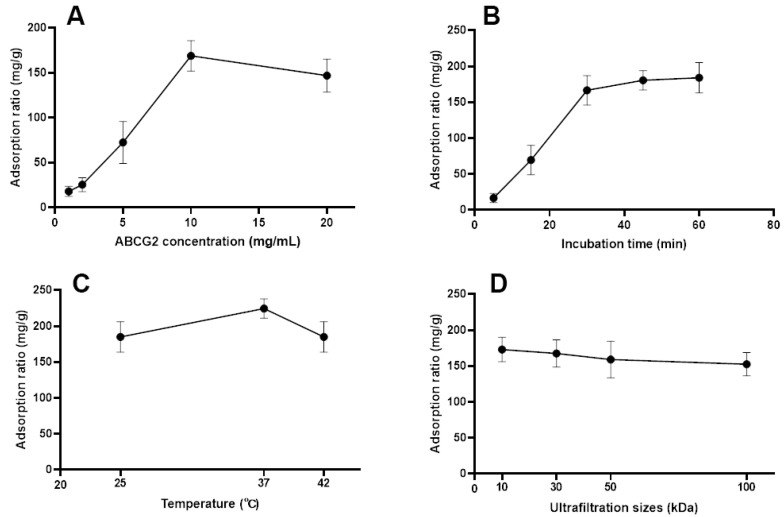
Impacts of parameters on interaction between the ligand and ABCG2. Impact of the ABCG2 concentration (**A**), incubation time (**B**), incubation temperature (**C**) and ultrafiltration size (**D**) on the binding ability between fumitremorgin C and ABCG2.

Upon incubation of the medicated plasma of Cortex Fraxini with ABCG2, protein-ligand complexes were formed. In order to achieve optimal screening performance with BA-UF-MS, it is necessary to investigate certain important parameters. The protein concentration of ABCG2 directly impacts the screening results. The results depicted in Figure 2A demonstrate that the binding quantity of fumitremorgin C exhibited an upward trend with increasing concentrations of ABCG2 up to 10 mg/mL, beyond which higher concentrations of ABCG2 had a detrimental impact. Furthermore, the duration of incubation influenced the binding quantity of ligands to target proteins. Figure 2B illustrates a substantial increase in the binding quantity of fumitremorgin C to ABCG2 from 5 min to 30 min, with no discernible changes observed when the incubation time reached 60 min, indicating the saturation of ABCG2 binding at the 30 min mark. Excessive incubation duration may result in a decline in protein activity, consequently leading to a diminished degree of binding [12]. The activity of ABCG2 is influenced by temperature. As depicted in Figure 2C, an incubation temperature of 37 °C exhibited the highest level of binding for fumitremorgin C. The size of the ultrafiltration tube played a significant role, thus necessitating an investigation of ultrafiltration sizes of 10, 30, 50 and 100 kDa. Figure 2D demonstrates that the 10 kDa ultrafiltration tube yielded the highest amount of ligand binding. In conclusion, the optimal screening conditions were determined to be as follows: ABCG2 concentration of 10 mg/mL, incubation time of 30 min, incubation temperature of 37 °C, and 10 kDa of ultrafiltration size.

### 2.3. Screen for ABCG2 Ligands Using BA-UF-MS

**Figure 3:**
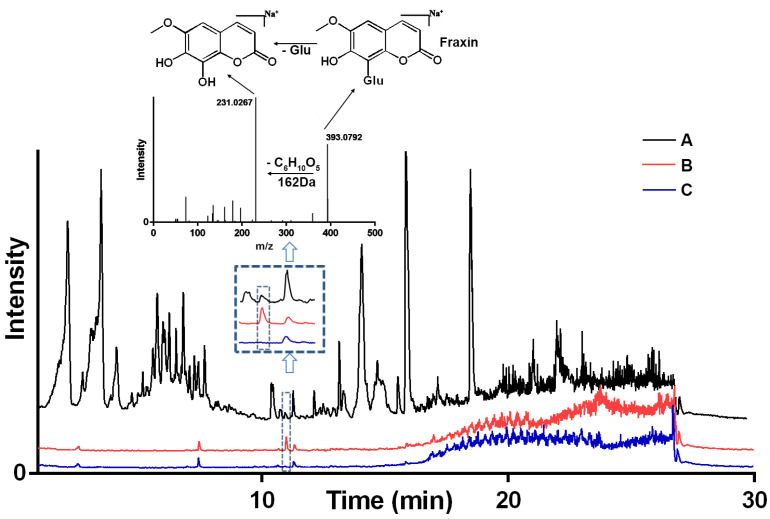
LC-MS chromatograms of the chemical constituents from Cortex Fraxini. (**A**) medicated plasma; (**B**) medicated plasma incubated with active ABCG2; (**C**) medicated plasma incubated with deactivated ABCG2. Glu: Glucose.

This experiment involves incubating ABCG2 with the medicated plasma of Cortex Fraxini, where ligands with a high affinity interact with the ABCG2 protein. Other components do not exhibit any specific interaction with ABCG2, and thorough washing steps are employed to eliminate low-affinity ligands. During the releasing phase, an organic solvent was employed to liberate binding ligands. Subsequently, the bound compounds were extracted using an organic solution and subjected to analysis via LC-MS. Utilizing the optimized conditions, medicated plasma was screened using BA-UF-MS, and the resulting chromatograms of medicated plasma, eluent solution from ABCG2, and a deactivated control sample solution are presented in Figure 3. By evaluating the peak area, a distinctive peak was observed in the chromatogram of the eluent solution, with a higher peak area compared to that of the control group, indicating favorable specificity. Based on the strategy principle, it was observed that this particular constituent possessed the ability to bind to ABCG2, thereby qualifying it as a potential ligand for ABCG2. The identification of potential active components was accomplished through the utilization of MS fragments and a self-constructed database comprising approximately 4500 compounds derived from natural herbs. Consequently, the compound fraxin was determined to be the hit compound.

### 2.4. Activity Test of the Hit Compound In Vitro and In Vivo

**Figure 4:**
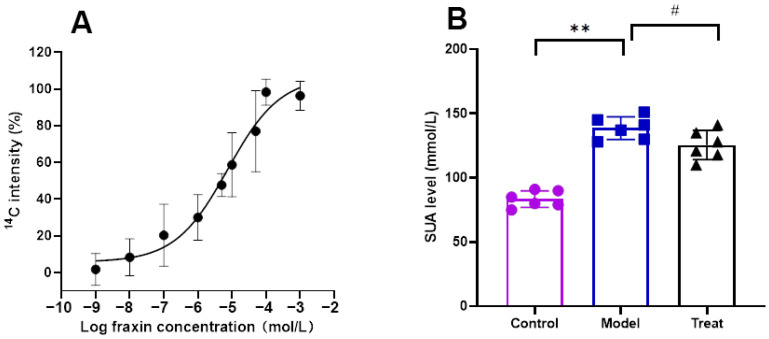
Activity evaluation of hit compound. (**A**) EC_50_ of fraxin for ABCG2-mediated ^14^C-UA uptake; (**B**) SUA-lowering activity of fraxin in model rats (# *p* < 0.05, ** *p* < 0.05).

It is important to note that the binding of a compound to a protein does not necessarily imply its role as an inhibitor or activator, as the compound may exhibit non-specific binding to non-functional sites of the protein [13]. The binding of a compound to a protein does not necessarily indicate whether it functions as an inhibitor or activator, as the compound may bind non-specifically to non-functional sites on the protein. Additional pharmacological investigation is required to determine the activating effect of the hit compound on ABCG2. The activity of fraxin was confirmed to activate ABCG2 in a concentration-dependent manner, as evidenced by the promotion of ABCG2 -mediated uptake of ^14^C -UA with an EC_50_ of 7.55 μM, as depicted in Figure 4A. To further validate its efficacy, fraxin was subsequently assessed for its ability to reduce SUA levels in rats. The findings of the study demonstrated that intragastrically administered fraxin led to a significant reduction in the level of SUA in rats (*p* < 0.05), as depicted in Figure 4B. These results were consistent with the outcomes of the target screening.

### 2.5. Affinity Verification between Fraxin and ABCG2

**Figure 5:**
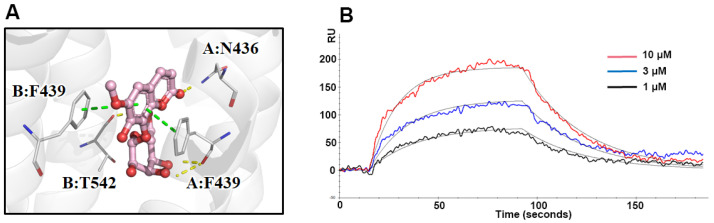
Affinity verification between fraxin and ABCG2. (**A**) Molecular docking analysis of ABCG2 and fraxin; (**B**) SPR assay of fraxin and ABCG2.

Molecular docking analysis was employed to investigate the binding sites and interaction between the hit compound and ABCG2. Fraxin exhibited a strong affinity towards the active pocket of ABCG2, as illustrated in Figure 5A, forming hydrogen bonds and π-π stacking with F439, F349, N436, and T542. The binding energy of fraxin with ABCG2 was calculated to be −13.145 kcal/mol. The docking results strongly suggested that fraxin has the potential to act as a ligand for ABCG2. To further validate the binding activity of ABCG2, the K_D_ value of fraxin was determined using SPR assay. The obtained result indicated that the K_D_ value for fraxin was calculated as 141.2 μM (Figure 5B).

## 3. Discussion

ABCG2 is a crucial urate transporter highly expressed in the epithelial cells of the intestine and kidney. This transporter plays a pivotal role in both renal urate overload and extra-renal urate underexcretion. Limited attention is currently devoted to UA-lowering drugs that target sites beyond the kidney. Additionally, numerous components found in TCMs exhibit low bioavailability when orally administered, resulting in limited absorption into the bloodstream and reaching the intended target organs. Conversely, certain components directly impact target sites within the intestine, such as by modulating the gut microbiota to reduce UA levels [14]. Given these considerations, we have selected ABCG2 as the protein target.

Commonly employed drugs for the treatment of hyperuricemia include benzbromarone, allopurinol, and febuxostat. Nevertheless, prolonged administration of these drugs is associated with various adverse effects, such as hepatotoxicity and nephrotoxicity [15]. Consequently, the identification of novel hyperuricemic drugs with reduced toxicity and fewer side effects has become a prominent research area in recent years. TCMs have been widely recognized as a valuable resource for identifying hit compounds in the field of drug discovery [16]. The diverse chemical structures present in these compounds offer a vast pool from which potential leads for drug development and optimization can be selected and refined. Considerable attention has been directed towards exploring the bioactive compounds derived from TCMs, as they hold promise as a novel and safer source for anti-hyperuricemia drug leads.

It is important to note that the pharmacological effects of TCMs primarily rely on the absorption of their components into the bloodstream. However, the oral administration of TCMs results in a significantly low transfer of these components into the blood. Moreover, the presence of numerous dietary and endogenous substances further complicates the identification and separation of the absorbed ingredients from TCMs. Given the intricate nature of the chemical constituents found in natural medicines and the minuscule concentration of their bioactive components within living organisms, the identification of potential target ligands from constituents absorbed into the bloodstream poses a formidable challenge [12]. Traditional approaches to isolating target protein ligands involve separation techniques and bioassay-guided fractionation. However, these conventional methods for screening bioactive compounds from complex systems are both time-consuming and labor-intensive [17]. Consequently, a targeted protein-oriented screening method would serve as an efficient strategy for the identification of active compounds from such intricate systems [18]. The utilization of target screens offers numerous benefits, primarily due to their enhanced speed and convenience in directly capturing target compounds, thereby eliminating the necessity for repetitive separation protocols of non-target analytes [19,20]. Consequently, the implementation of the BA-UF-MS strategy is anticipated to expedite the process of natural-product based drug discovery.

Certain parameters, such as protein concentration, incubation time, incubation temperature, and the size of the ultrafiltration tube depend on the characteristics of ABCG2 and the structure of ligands. The characteristics of ABCG2 are the main factor that influences protein-ligand interactions, due to the ligand structure beingvariable. Fumitremorgin C, a potent inhibitor, has been identified as a specific ligand for ABCG2. Consequently, it has been selected as the focal point for conditional optimization in order to potentially uncover other bioactive ligands similar to Fumitremorgin C. The correlation between the molecular weight of a protein and the filter size of an ultrafiltration membrane is not absolute. Moreover, the observed molecular size of ABCG2 (72 kDa) in a buffer solution surpasses its theoretical value, and protein interactions can lead to comparable outcomes in ultrafiltration membranes with filter sizes of 50 kDa and 100 kDa.

Cortex Fraxini has been utilized in the management of hyperuricemia and gout, thus presenting a promising opportunity to identify potential therapeutic components from Cortex Fraxini that target hyperuricemia. The assessment of a compound’s binding ability to a specific protein is essential in evaluating the biological effects of TCMs [21]. Notably, the Cortex Fraxini extract demonstrated significant activity in ^14^C-UA uptake experiments, indicating the presence of potential ABCG2 activated ligands within Cortex Fraxini. Employing the BA-UF-MS strategy, fraxin was screened and confirmed to exhibit activity both in vitro and in vivo, thereby suggesting its potential as an anti-hyperuricemia agent [22].

The identification of ligand compounds primarily depends on their comparison with databases containing natural product standard substances. During the screening process, we analyze and compare the mass spectrometry data, focusing on the most significant specific alterations. The compound exhibiting the highest degree of similarity is determined as fraxin. Furthermore, the compound’s structure is confirmed by examining the fragmentation pattern of its mass spectrometry fragments. However, the utilization of lower electron impact energy in the mass spectrometry limits the acquisition of additional secondary fragmentation information. In addition to fraxin, several candidate hit compounds identified in the screening results were not included in this study, necessitating further investigation into their potential for reducing uric acid levels. Moreover, the limitations of the mass spectrometry standard substance library restrict its ability to identify entirely novel hit compounds with unique structural characteristics.

To explore the interaction between the hit compound and ABCG2, we employed molecular docking and SPR assay. Molecular docking, a virtual method influenced by force field, temperature, and pH, provides a theoretical understanding of the receptor-ligand interaction [23]. To complement this, we conducted real experiments and determined the binding affinity between fraxin and ABCG2 to be 141.2 μM using SPR assay. These findings substantiate the mechanistic perspective and validate the accuracy and reliability of the BA-UF-MS strategy.

## 4. Materials and Methods

### 4.1. Reagents

Chemical compounds of fumitremorgin C and fraxin were produced from MedChemExpress (Shanghai, China). Cortex Fraxini (Lot: 220509) was purchased from the GuoDa drugstore (Shanghai, China), which was obtained from *Fraxinus rhynchophylla Hance* in Shanxi province, China. HPLC-grade reagents were obtained from Fisher Scientific (Fair Lawn, NJ, USA). All other chemicals were bought from standard commercial sources. Recombinant ABCG2 was purchased from Feiyue Biotechnology Co., Ltd. (Wuhan, China), and centrifugal ultrafiltration filters with a cutoff membrane were provided by Merck millipore Co., Ltd. (Billerica, MA, USA). ^14^C-uric acid was purchased from American Radiolabeled Chemicals (St. Louis, MO, USA). Membrane vesicles obtained from ABCG2-overexpressing cells were purchased from SOLVO Biotechnology Biotechnology (Budapest, Hungary).

### 4.2. Preparation of the Cortex Fraxini Extract

The powdered Cortex Fraxini (1.0 kg) was subjected to ultrasonic extraction using 10 L of 75% ethanol and a power of 500 W for a duration of 1 h. The resulting extracting solution was then centrifuged at a speed of 4000 rpm for a duration of 3 min. Subsequently, the filtrate was concentrated under reduced pressure at a temperature of 50 °C. The remaining solution was then subjected to lyophilization in order to obtain a freeze-dried powder of Cortex Fraxini extract.

### 4.3. Preparation of Medicated Plasma of Cortex Fraxini

A total of 6 male Sprague Dawley rats, weighing 200 g, were procured from the Experimental Animal Center of Xuzhou Medical University in Xuzhou, China. All experimental procedures were conducted in accordance with the guidelines and regulations set forth by the Medical Ethic Committee of Xuzhou Medical University (NO. L20210226100). The prepared Cortex Fraxini extract, suspended in 0.5% CMC-Na, was administered to the rats via intragastric gavage at a dose of 1.7 g/kg body weight [24]. Blood samples were collected via retro-orbital bleeding at specific time intervals (0.5, 1, 3, 6, 12, and 24 h). Approximately 0.5 mL of blood was collected at each time point and immediately centrifuged at 13,000 rpm for 10 min. The resulting plasma samples from 6 rats were combined and stored at −80 °C until further analysis.

### 4.4. Screen of Potential ABCG2 Ligands with BA-UF-MS

The method employed for analysis involved three steps: loading, washing, and releasing [25]. The ABCG2 solution (5 μg/mL) was prepared using a phosphate buffer solution (PBS, 100 mM, pH 7.4). Subsequently, 100 μL of medicated plasma and 100 μL of ABCG2 solution were combined in an EP tube and incubated at 37 °C for 30 min. Following incubation, the formed ABCG2-ligand complexes were filtered through a 10 kDa ultrafiltration (UF) membrane. The target-ligand complexes were retained by centrifugation at 13,000 rpm for 10 min, while unbound compounds in the UF tube were eliminated through 6 rounds of washing with 200 μL of PBS and subsequent centrifugation. Unbound compounds successfully traversed the UF membrane, whereas ligands bound to the ABCG2 protein did not. Consequently, the ABCG2-ligand complexes were isolated and acquired. Subsequently, the attached ligands were liberated by subjecting them to ultrasonication and centrifugation at 13,000 rpm for 10 min, repeated three times. The filtrates from each sample were consolidated for subsequent BA-UF-MS analysis.

To address the issue of nonspecific binding in the ligand screening process, the ABCG2 protein was subjected to denaturation by boiling it in water for a duration of one hour. A control group was established by co-incubating the denatured protein with medicated plasma, followed by conducting the subsequent screening procedure in a similar manner. Subsequently, liquid chromatography, coupled with high-resolution mass spectrometry (LC-HRMS, Orbitrap Exploris 120, Thermo Fisher Scientific, Waltham, MA, USA), was employed to analyze and identify compounds that specifically target ABCG2 within the medicated plasma. By comparing the peak areas of LC-HRMS chromatograms obtained from the experimental and control samples (representing inactive ABCG2), potential ABCG2 ligands were identified and screened within the medicated plasma.

LC-HRMS conditions for compound analysis of Cortex Fraxini were listed as follows. LC analysis was conducted using an UHPLC system equipped with a C18 column (Waters UPLC BEH, 1.7 μm, 2.1 × 100 mm). The flow rate was set at 0.4 mL/min, and the sample injection volume was 5 μL. The mobile phase consisted of 0.1% formic acid in water (A) and 0.1% formic acid in acetonitrile (B). The elution gradient program involved multiple linear steps as follows: 0–3.5 min, 95–85% A; 3.5–6 min, 85–70% A; 6–6.5 min, 70–70% A; 6.5–12 min, 70–30% A; 12–12.5 min, 30–30% A; 12.5–18 min, 30–0% A; 18–25 min, 0–0% A; 25–26 min, 0–95% A; 26–30 min, 95–95% A. The MS and MS/MS data were obtained using a mass spectrometer coupled with Xcalibur software (v 4.2), employing the IDA acquisition mode. The mass range during each acquisition cycle was set from 100 to 1500, with the top four ions screened and their corresponding MS/MS data acquired. The sheath gas flow rate was 30 Arb, the aux gas flow rate was 10 Arb, the ion transfer tube temperature was set at 350 °C, and the vaporizer temperature was also set at 350 °C. The full MS resolution was set at 60,000, while the MS/MS resolution was set at 15,000. Collision energy was applied at 16/38/42 in NCE mode, and the spray voltage was set at 5.5 kV (positive).

### 4.5. Identification of Potential ABCG2 Ligands with LC-HRMS

The identification of potential active components was accomplished through the utilization of MS fragments and a self-constructed database, comprising approximately 4500 compounds derived from natural herbs. The database encompasses a wide range of chemical compounds, including more than 570 distinct alkaloids (such as piperidine alkaloids, quinoline alkaloids, pyridine alkaloids, and quinoline alkaloids), 800 varieties of flavonoids (including isoflavonoids, flavones, dihydrochalcones, and dihydroflavonoids), 1100 types of terpenes (including cyclic sesquiterpenes, lavenderane terpenes, and cyclopentane-cycloene ether terpenes), 360 types of phenylpropanoids (including coumarins, phenylpropanoic acids, and lignans), 50 types of steroids, 60 types of quinones, over 90 types of organic acids, and various other components. The identification of components present in Cortex Fraxini can be achieved by comparing their mass information with a database. Furthermore, the utilization of fragmentation patterns from secondary mass spectrometry can aid in the process of identification.

### 4.6. Optimization of Experimental Parameters

In order to attain the most favorable screening outcomes, various factors that have the potential to influence the results were examined. These factors encompassed the concentration of ABCG2 (ranging from 1 to 20 mg/mL), the duration of incubation (ranging from 5 to 60 min), the temperature of incubation (25, 37, and 42 °C), and the size of ultrafiltration filters (ranging from 10 to 100 kDa). Fumitremorgin C (a known ligand of ABCG2) was selected as the representative compounds to optimize the screening conditions. The adsorption ratio (mg/g) of fumitremorgin C was used to evaluate optimization procedure, which was calculated as m_a_/m_0_, where m_a_ (mg) represented the mass of adsorptive and subsequently eluted fumitremorgin C, and m_0_ (mg) represented the mass of added fumitremorgin C for incubation.

### 4.7. Study of ^14^C-UA Uptake by Membrane Vesicles

The evaluation of the ^14^C-UA uptake of Cortex Fraxini extract or hit compounds was conducted using membrane vesicles obtained from ABCG2-overexpressing cells [26]. In 96-well plates, membrane vesicles, ^14^C-UA, and a hit compound solution were combined and incubated with PBS buffer for a duration of 30 min at a temperature of 37 °C. The uptake reactions of ^14^C-UA were initiated by the addition of MgATP, and the membrane vesicles were subsequently washed three times with ice-cold DPBS (Dulbecco’s phosphate-buffered saline) in order to terminate the reaction after a period of 5 min. Following filtration through glass fiber filters, the filters were washed three times with ice-cold DPBS. The quantity of substrate present within the filtered vesicles was determined by measuring intracellular radioactivity using a liquid scintillation counter (PerkinElmer, Waltham, MA, USA) subsequent to the addition of scintillant. Each treatment was measured in triplicate.

### 4.8. Study of SUA-Lowing Effect of the Hit Compound in Hyperuricemic Rats

The hit compound was assessed in a rat model of acute hyperuricemia induced by hypoxanthine and potassium cyanate [27]. Six SD rats were allocated to each of the following groups: control, hyperuricemic model, and treatment. The hyperuricemic model group rats were administered xanthine intragastrically and potassium oxonate subcutaneously. The treatment group rats were orally administered 10 mg/kg of the hit compound (dissolved in 0.5% CMC-Na) 3 h prior to xanthine and potassium oxonate administration. Blood samples were collected via retro-orbital bleeding after 6 h of hyperuricemia induction. The levels of SUA were assessed using the UA ELISA kit (Bioroyee Co., Ltd., Beijing, China).

### 4.9. Molecular Docking

Molecular docking was conducted to simulate the process of molecular recognition and determine the binding energies between the hit compound and ABCG2 using PyMOL v1.3 software. The 3D crystal structure of ABCG2 from mitoxantrone (6VXI) was obtained from the Research Collaboratory for Structural Bioinformatics Protein Data Bank (RCSB PDB). The 3D chemical structures of ligand compounds were acquired either from the PubChem website or generated using the ChemDraw software package Pro 12.0. The ligands and ABCG2 were prepared and processed for minimization, and subsequently saved in pdbqt format. Following the selection of specific residues within the pocket region, a CHARMm force field was applied to the ligands and ABCG2 after targeting the grid box [28,29,30].

### 4.10. Surface Plasmon Resonance Analysis

The local surface plasmon resonance (LSPR) experiment was performed using an OpenSPR (NicoyaLifesciences, Waterloo, ON, Canada) for the analysis of SPR [31]. The COOH sensor chip was utilized for capture-coupling to immobilize ABCG2 protein (50 μg/mL), and the interaction between ABCG2 and the hit compound was observed at 37 °C. The running buffer utilized in this study was a PBS buffer containing 5% DMSO. Solutions of varying concentrations of the hit compound were sequentially flowed through the chips at a rate of 20 μL/min. Simultaneously, running buffer correction was conducted.

### 4.11. Statistical Analysis

All experimental data were analyzed using SPSS software (19.0) and recorded as mean ± standard deviation for three replicates. Statistical significance was determined at a threshold of *p* < 0.05.

## 5. Conclusions

In the current investigation, fraxin was identified from medicated plasma of Cortex Fraxini using a rapid and efficient BA-UF-MS strategy. The bioactivity of fraxin was assessed through in vitro and in vivo experiments, including determination of the EC50 value, evaluation of its SUA lowering effect, analysis of molecular docking scores, and determination of the affinity constant K_D_. The findings unequivocally established that fraxin possesses promising bioactivity in reducing UA levels, making it a potential candidate for the treatment of hyperuricemia and gout. In summary, the findings of this study indicate that medicated plasma derived from TCM holds promise as a valuable resource for identifying potential hit compounds. Furthermore, the BA-UF-MS approach proves to be an effective strategy for the discovery of active ingredients from biological samples.

## Data Availability

The data presented in this study are available on request from the corresponding author.

## References

[1] Johnson R.J., Bakris G.L., Borghi C., Chonchol M.B., Feldman D., Lanaspa M.A., Merriman T.R., Moe O.W., Mount D.B., Lozada L.G.S. (2018). Hyperuricemia, Acute and Chronic Kidney Disease, Hypertension, and Cardiovascular Disease: Report of a Scientific Workshop Organized by the National Kidney Foundation. Am. J. Kidney Dis..

[2] Bardin T., Richette P. (2014). Definition of hyperuricemia and gouty conditions. Curr. Opin. Rheumatol..

[3] Lanaspa M.A., Andres-Hernando A., Kuwabara M. (2020). Uric acid and hypertension. Hypertens. Res..

[4] Hoque K.M., Dixon E.E., Lewis R.M., Allan J., Gamble G.D., Phipps-Green A.J., Halperin Kuhns V.L., Horne A.M., Stamp L.K., Merriman T.R. (2020). The ABCG2 Q141K hyperuricemia and gout associated variant illuminates the physiology of human urate excretion. Nat. Commun..

[5] Woodward O.M., Kottgen A., Coresh J., Boerwinkle E., Guggino W.B., Kottgen M. (2009). Identification of a urate transporter, ABCG2, with a common functional polymorphism causing gout. Proc. Natl. Acad. Sci. USA.

[6] Stiburkova B., Pavelcova K., Pavlikova M., Jesina P., Pavelka K. (2019). The impact of dysfunctional variants of ABCG2 on hyperuricemia and gout in pediatric-onset patients. Arthritis Res. Ther..

[7] Huls M., Brown C.D., Windass A.S., Sayer R., van den Heuvel J.J., Heemskerk S., Russel F.G., Masereeuw R. (2008). The breast cancer resistance protein transporter ABCG2 is expressed in the human kidney proximal tubule apical membrane. Kidney Int..

[8] Li Q., Lin H., Niu Y., Liu Y., Wang Z., Song L., Gao L., Li L. (2020). Mangiferin promotes intestinal elimination of uric acid by modulating intestinal transporters. Eur. J. Pharmacol..

[9] Lu Y.H., Chang Y.P., Li T., Han F., Li C.J., Li X.Y., Xue M., Cheng Y., Meng Z.Y., Han Z. (2020). Empagliflozin Attenuates Hyperuricemia by Upregulation of ABCG2 via AMPK/AKT/CREB Signaling Pathway in Type 2 Diabetic Mice. Int. J. Biol. Sci..

[10] Wang Y., Zhao M., Xin Y., Liu J., Wang M., Zhao C. (2016). ^1^H NMR and MS based metabolomics study of the therapeutic effect of Cortex Fraxini on hyperuricemic rats. J. Ethnopharmacol..

[11] Song H.P., Chen J., Hong J.Y., Hao H., Qi L.W., Lu J., Fu Y., Wu B., Yang H., Li P. (2015). A strategy for screening of high-quality enzyme inhibitors from herbal medicines based on ultrafiltration LC-MS and in silico molecular docking. Chem. Commun..

[12] Dong X., Wang B., Cao J., Zheng H., Ye L.H. (2021). Ligand fishing based on bioaffinity ultrafiltration for screening xanthine oxidase inhibitors from citrus plants. J. Sep. Sci..

[13] Li L., Kong J., Yao C.H., Liu X.F., Liu J.H. (2019). Rapid identification of urokinase plasminogen activator inhibitors from Traditional Chinese Medicines based on ultrafiltration, LC-MS and in silico docking. J. Pharm. Biomed. Anal..

[14] Sun X., Wen J., Guan B., Li J., Luo J., Li J., Wei M., Qiu H. (2022). Folic acid and zinc improve hyperuricemia by altering the gut microbiota of rats with high-purine diet-induced hyperuricemia. Front. Microbiol..

[15] White W.B., Saag K.G., Becker M.A., Borer J.S., Gorelick P.B., Whelton A., Hunt B., Castillo M., Gunawardhana L., CARES Investigators (2018). Cardiovascular Safety of Febuxostat or Allopurinol in Patients with Gout. N. Engl. J. Med..

[16] Zhang H., Xu C., Tian Q., Zhang Y., Zhang G., Guan Y., Tong S., Yan J. (2021). Screening and characterization of aldose reductase inhibitors from Traditional Chinese medicine based on ultrafiltration-liquid chromatography mass spectrometry and in silico molecular docking. J. Ethnopharmacol..

[17] Gaudencio S.P., Pereira F. (2015). Dereplication: Racing to speed up the natural products discovery process. Nat. Prod. Rep..

[18] Ciesla L., Moaddel R. (2016). Comparison of analytical techniques for the identification of bioactive compounds from natural products. Nat. Prod. Rep..

[19] Wei H., Zhang X., Tian X., Wu G. (2016). Pharmaceutical applications of affinity-ultrafiltration mass spectrometry: Recent advances and future prospects. J. Pharm. Biomed. Anal..

[20] Chen G., Huang B.X., Guo M. (2018). Current advances in screening for bioactive components from medicinal plants by affinity ultrafiltration mass spectrometry. Phytochem. Anal..

[21] Arai M.A., Ishikawa N., Tanaka M., Uemura K., Sugimitsu N., Suganami A., Tamura Y., Koyano T., Kowithayakorn T., Ishibashi M. (2016). Hes1 inhibitor isolated by target protein oriented natural products isolation (TPO-NAPI) of differentiation activators of neural stem cells. Chem. Sci..

[22] Li J.M., Zhang X., Wang X., Xie Y.C., Kong L.D. (2011). Protective effects of cortex fraxini coumarines against oxonate-induced hyperuricemia and renal dysfunction in mice. Eur. J. Pharmacol..

[23] Zhu C., Niu H., Nie A., Bian M. (2021). Bioactivity-guided separation of potential alpha-glycosidase inhibitor from clerodendranthus spicatus based on HSCCC coupled with molecular docking. Sci. Rep..

[24] Wang Y., Zhao M., Ou Y., Zeng B., Lou X., Wang M., Zhao C. (2016). Metabolic profile of esculin in rats by ultra high performance liquid chromatography combined with Fourier transform ion cyclotron resonance mass spectrometry. J. Chromatogr. B Analyt. Technol. Biomed. Life Sci..

[25] Luo S., Guo L., Sheng C., Zhao Y., Chen L., Li C., Jiang Z., Tian H. (2020). Rapid identification and isolation of neuraminidase inhibitors from mockstrawberry (*Duchesnea indica* Andr.) based on ligand fishing combined with HR-ESI-Q-TOF-MS. Acta Pharm. Sin. B.

[26] Taniguchi T., Ashizawa N., Matsumoto K., Saito R., Motoki K., Sakai M., Chikamatsu N., Hagihara C., Hashiba M., Iwanaga T. (2019). Pharmacological Evaluation of Dotinurad, a Selective Urate Reabsorption Inhibitor. J. Pharmacol. Exp. Ther..

[27] Zhao T., Meng Q., Sun Z., Chen Y., Ai W., Zhao Z., Kang D., Dong Y., Liang R., Wu T. (2020). Novel Human Urate Transporter 1 Inhibitors as Hypouricemic Drug Candidates with Favorable Druggability. J. Med. Chem..

[28] Akacha A., Badraoui R., Rebai T., Zourgui L. (2022). Effect of extract on methotrexate-induced testicular injury: A biochemical, docking and histological study. J. Biomol. Struct. Dyn..

[29] Alreshidi M., Abdulhakeem M.A., Badraoui R., Amato G., Caputo L., De Martino L., Nazzaro F., Fratianni F., Formisano C., De Feo V. (2023). *Pulicaria incisa* (Lam.) DC. as a Potential Source of Antioxidant, Antibacterial, and Anti-Enzymatic Bioactive Molecules: Phytochemical Constituents, In Vitro and In Silico Pharmacological Analysis. Molecules.

[30] Ben Saad H., Frikha D., Bouallegue A., Badraoui R., Mellouli M., Kallel H., Pujo J.M., Ben Amara I. (2023). Mitigation of Hepatic Impairment with Polysaccharides from Red Alga *Albidum corallinum* Supplementation through Promoting the Lipid Profile and Liver Homeostasis in Tebuconazole-Exposed Rats. Pharmaceuticals.

[31] Hou Y., Che D., Wei D., Wang C., Xie Y., Zhang K., Cao J., Fu J., Zhou N., He H. (2019). Phenothiazine antipsychotics exhibit dual properties in pseudo-allergic reactions: Activating MRGPRX2 and inhibiting the H_1_ receptor. Mol. Immunol..

